# Bio-informatics assessment schema (BIAS) to improve myocardial perfusion image diagnostic and prognostic value: the NHLBI-sponsored women's ischemia syndrome evaluation (WISE) study

**DOI:** 10.1186/1532-429X-16-S1-P201

**Published:** 2014-01-16

**Authors:** Mark Doyle, Gerald Pohost, Leslee J Shaw, Diane V Thompson, Sheryl F Kelsey, B Delia Johnson, William J Rogers, Geetha Rayarao, Barry L Sharaf, Carl J Pepine, C Noel Bairey Merz, Robert W Biederman

**Affiliations:** 1 Cardiac MRI, Allegheny General Hospital, Pittsburgh, Pennsylvania, USA; 2 Cardiology, University of Pittsburgh, Pittsburgh, Pennsylvania, USA; 3 Cardiology, Cedars-Sinai Medical Ctr, Los Angeles, California, USA; 4 Cardiology, Univ. of Alabama, Birmingham, Alabama, USA; 5 Cardiology, Brown University, Providence, Rhode Island, USA; 6 Cardiology, University of Florida, Gainesville, Florida, USA; 7 Cardiology, Univ. of Southern California, Los Angeles, California, USA; 8 Cardiology, Atlanta Cardiovascular Research Inst., Atlanta, Georgia, USA

## Background

Introduction: When assessing myocardial perfusion image (MPI) data for diagnosis or prediction of prognosis it is common to evaluate the technology using a Receiver-Operator Characteristic (ROC) curve. The diagonal response represents the knowledge prior to conducting the test (i.e. the diagonal represents random chance). We present an approach termed Bio-Informatics Assessment Schema (BIAS) that provides an elevated baseline from which to evaluate the MPI reading, i.e. indicating that at baseline, more data is available than random chance. Here we describe how the BIAS formulae are generated for cardiovascular magnetic resonance image (CMRI) data applied to the Women's Ischemia Syndrome Evaluation (WISE) Study.

## Methods

Methods: Women (n = 182), mean age 59 ± 11 yrs, with symptoms suggestive of myocardial ischemia underwent MPI and cardiac function evaluation separately by CMRI and Single Positron Computed Tomography (SPECT) and were followed (40 ± 17 months) to document MACE (CV death, myocardial infarction, and hospitalization for congestive heart failure). Abnormal perfusion defects were noted for each MPI modality using clinical criteria (clinical reading) and if at least one region was abnormal, the study was considered to be positive for disease. Multiple linear regression models were generated, each predicting MPI status from one modality (e.g. CMRI) using data from a second modality (e.g. SPECT). The BIAS models were generated by removing the myocardial perfusion status from the linear regression models, i.e. the BIAS equations did not contain any knowledge of the MPI status (e.g. BIAS model = -.43 + end systolic volume index × 0.011 + average myocardial wall thickness × 0.037). The area under the curve (AUC) for ROC analysis was calculated for the natural MPI status of the CMRI data and for the BIAS curve. ROC analysis was conducted for detection of obstructive coronary artery disease (CAD≥50% stenosis) and prediction of MACE.

## Results

**Figure 1:**
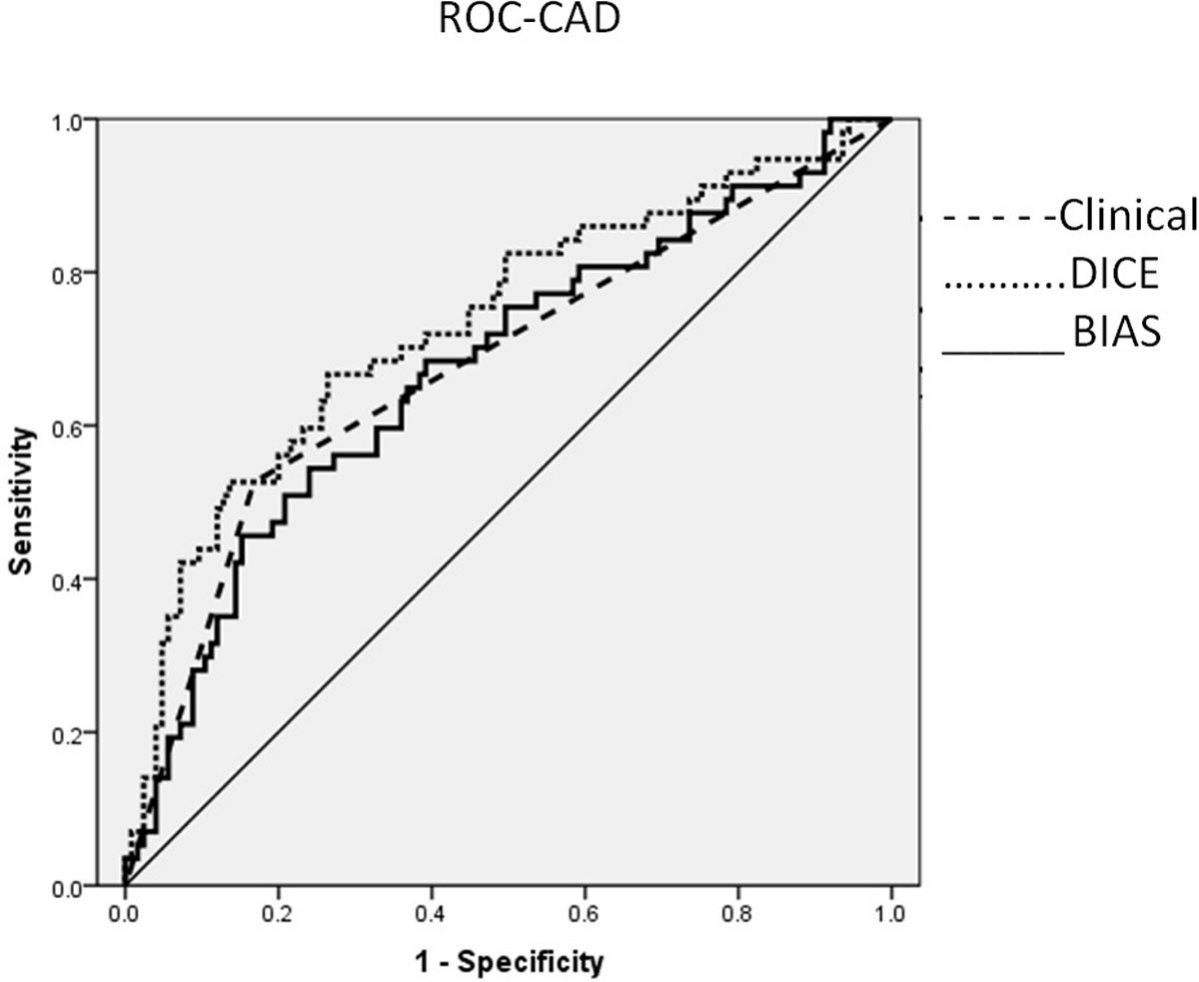
**ROC of CAD Detection**.

**Figure 2:**
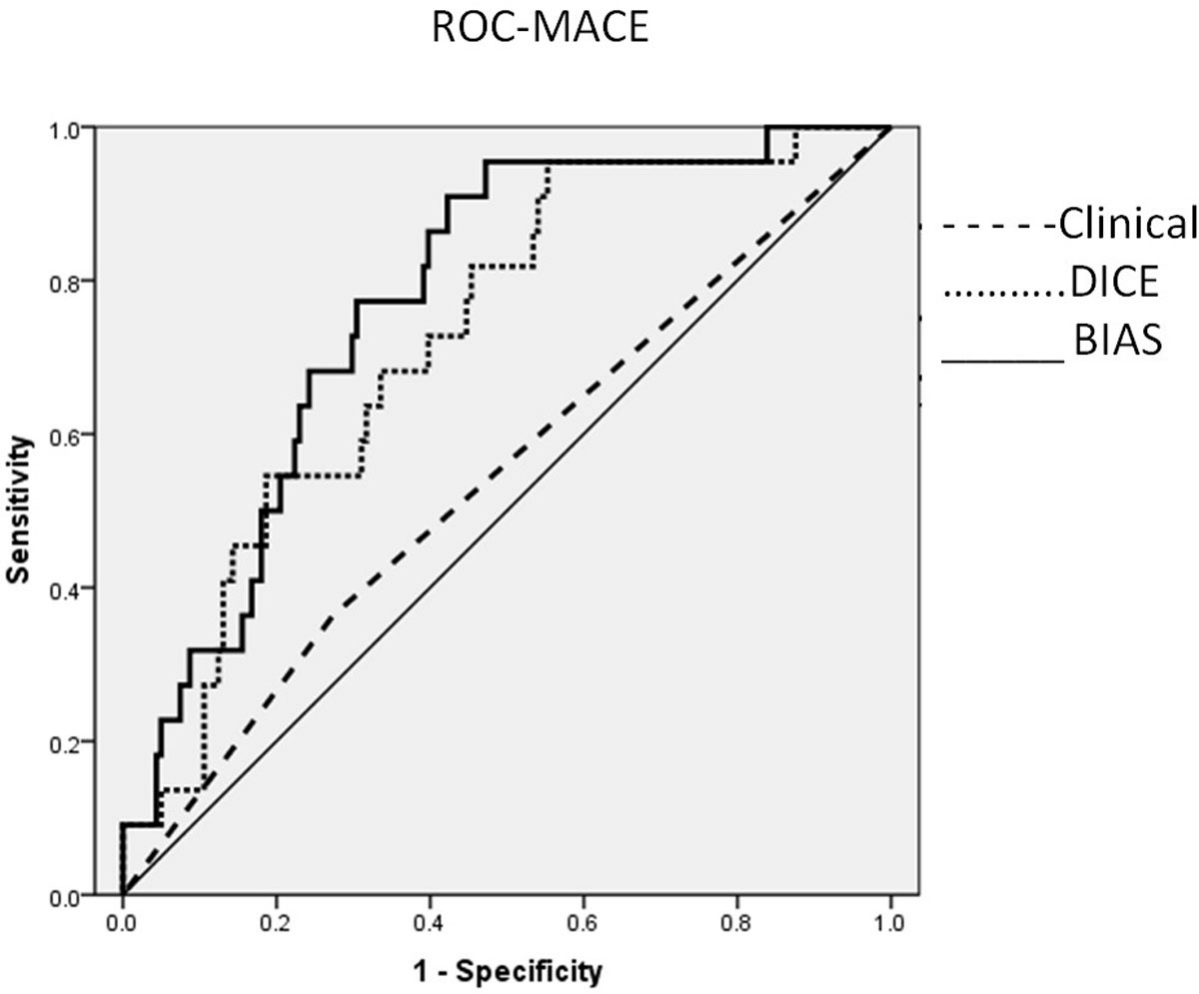
**ROC of MACE Detection**.

Results: CAD was present in 57 (31%) and MACE occurred in 22 women (12%). For prediction of CAD, the AUCs were: clinical MPI reading 0.68 and BIAS reading 0.67 (Figure 1). For prediction of MACE, the AUCs were: clinical MPI reading 0.54 and BIAS reading 0.77 (Figure 2).

## Conclusions

Conclusions: BIAS modeling, incorporating cardiac variables measured by CMRI, produced an elevated baseline on ROC analysis (diagnostic and prognostic) that was higher than the clinical MPI reading in prognostic case. The significance of this is that this elevated baseline represents the information status prior to reading the MPI data. The next logical step is to incorporate this baseline knowledge into reading the MPI status, which is expected to result in dramatic improvement of MPI data.

## Funding

This work was supported by contracts from the National Heart, Lung and Blood Institutes nos. N01-HV-68161, N01-HV-68162, N01-HV-68163, N01-HV-68164, grants U0164829, U01 HL649141, U01 HL649241, T32HL69751, R01 HL090957, 1R03AG032631 from the National Institute on Aging, GCRC grant MO1-RR00425 from the National Center for Research Resources, the National Center for Advancing Translational Sciences Grant UL1TR000124, and grants from the Gustavus and Louis Pfeiffer Research Foundation, Danville, NJ, The Women's Guild of Cedars-Sinai Medical Center, Los Angeles, CA, The Ladies Hospital Aid Society of Western Pennsylvania, Pittsburgh, PA, and QMED, Inc., Laurence Harbor, NJ, the Edythe L. Broad Women's Heart Research Fellowship, Cedars-Sinai Medical Center, Los Angeles, California, the Barbra Streisand Women's Cardiovascular Research and Education Program, Cedars-Sinai Medical Center, Los Angeles and The Society for Women's Health Research (SWHR), Washington, D.C.

